# Insights into the Mycosphere Fungal Community and Its Association with Nucleoside Accumulation in *Ophiocordyceps sinensis*

**DOI:** 10.3390/jof11100696

**Published:** 2025-09-25

**Authors:** Jianshuang Zhang, Wen Zhang, Xiaodan Wu, Weidi Fu, Chaoyun Yang, Nana Long

**Affiliations:** School of Life Sciences, Guizhou Normal University, Guiyang 550025, China

**Keywords:** *Ophiocordyceps sinensis*, soil fungi, diversity, nucleosides, species composition

## Abstract

Soil microbiomes are critical environmental factors influencing the occurrence and quality formation of *Ophiocordyceps sinensis*, a valuable medicinal fungus endemic to the Qinghai-Tibet Plateau. However, few studies have explored the relationship between mycosphere soil fungal communities and the quality characteristics of *O. sinensis.* This research aimed to systematically analyze the structural characteristics and diversity of fungal communities in mycosphere soils of *O. sinensis* from eight geographical regions using Illumina high-throughput sequencing, and compare the nucleoside contents in *O. sinensis* from the corresponding sites. Alpha diversity indices showed that soil samples from Yushu and Guoluo in Qinghai Province exhibited higher fungal richness and diversity compared to other regions, whereas samples from Qamdo in Tibet showed the lowest diversity. Beta diversity analysis indicated significant differences in fungal community structure across various regions (*R* = 0.861, *p* = 0.001). At the phylum level, Ascomycota overwhelmingly dominated throughout all mycosphere soils of *O. sinensis* (96.30–99.88%), followed by Mortierellomycetes (0.25–2.25%). Network analysis revealed that *Ophiocordyceps* emerged as the core taxon in the mycosphere fungal communities, suggesting its central role in shaping the structure of the fungal networks. Additionally, *O. sinensis* from Yushu contained the highest total nucleoside content, indicating that the accumulation of nucleosides in *O. sinensis* may be affected by the composition of mycosphere soil fungi. Furthermore, correlation analysis indicated a significant positive relationship between several mycosphere fungal abundances and nucleoside accumulation in *O. sinensis*, such as *Naganishia*, *Acicuseptoria*, *Nectria*, *Serendipita*, and *Humicola*. These findings would provide a theoretical foundation for improving artificial cultivation strategies of *O. sinensis*.

## 1. Introduction

*Ophiocordyceps sinensis*, commonly known as Chinese cordyceps, is a parasitic entomopathogenic fungus endemic to the alpine regions of the Qinghai-Tibet Plateau. It infects larvae of insects in the Hepialidae family and develops into a distinctive biological complex consisting of the fungal stroma and the mummified host larva. Owing to its high medicinal value and increasing commercial demand, wild populations have been extensively harvested over the past decades, raising serious concerns about ecological sustainability of this species [1]. Artificial cultivation has therefore been explored as an alternative to relieve harvesting pressure [2]. Despite some progress, cultivation remains challenging due to low infection success rates and unstable quality. These limitations have been proposed to be closely related to soil microbial communities, which constitute an essential part of the natural habitats of *Cordyceps* fungi [3,4].

Soil fungi play a crucial role in mushroom primordia formation and development, as they not only decompose organic matter but also engage in species-specific interactions that modulate nutrient cycling and carbon retention [5,6]. Previous study demonstrated that the phyla Ascomycota, Basidiomycota, and Mortierellomycota were dominant in the mycosphere soils of *O. sinensis*, highlighting a stable yet complex fungal ecological structure [7]. In addition, a wide range of entomopathogenic and phytopathogenic fungi, including species of *Cryptococcus*, *Mortierella*, and *Isaria* genera, have been detected using both high-throughput sequencing and culture-dependent approaches, suggesting potential synergistic or competitive interactions with *O. sinensis* [8]. Interestingly, Shao et al. [9] reported that higher fungal diversity in the soil habitat might suppress the occurrence of *O. sinensis*, implying that community complexity could regulate host infection dynamics. Furthermore, a recent study revealed that there were significant differences in fungal community composition between mycosphere soils of artificially cultivated and wild *O. sinensis* [3]. Together, these findings suggest that the domestication, infection processes, and stroma development of *O. sinensis* are ecologically linked to the mycosphere fungal community, which may thereby affect its growth and quality. However, previous investigations have generally been restricted to single or a few production sites, which limits the ecological inference that can be drawn. A broader geographical investigation is therefore needed to clarify whether patterns of fungal diversity are consistently associated with regional differences in the quality of *O. sinensis.*

The quality formation of *O. sinensis* is closely associated with the accumulation of diverse bioactive constituents, among which nucleosides represent one of the main groups [10]. Several nucleosides, including adenosine, uridine, cytidine, guanosine, and inosine, have been considered as quality indicators for *O. sinensis* due to their important roles in regulating central nervous system functions, anti-inflammatory, and anti-tumor effects [11,12]. It is known that nucleosides accumulation in *O. sinensis* varied significantly depending on strain type, developmental stage, and geographical origin [13]. Recent studies indicated that microorganisms inhabiting the same niches could modulate the production of bioactive metabolites in macrofungi and plants through nutrient competition, antagonism, or signaling interactions [14,15,16]. For instance, *Streptomyces thermoviolaceus* was reported to enhance the crude protein, crude polysaccharide, total amino acid, and essential amino acid accumulation in oyster mushrooms [17]. Research on *C. takaomontana*, a closely related species of *O. sinensis*, revealed that two native soil fungi of *Fusarium paeoniae* and *Bjerkandera minispora* significantly increased its antioxidant enzyme activities and total triterpenoid levels [18]. Collectively, these findings suggest that microhabitat fungi may exert substantial influence on the nutritional and bioactive composition of *O. sinensis*. However, the specific relationship between mycosphere fungal community diversity and the nucleoside accumulation in *O. sinensis* remains poorly understood, representing a critical knowledge gap that warrants systematic investigation.

In the present work, we hypothesized that fungal communities in the mycosphere of *O. sinensis* exhibit distinct regional variations, and these shifts are closely linked to differences in nucleoside accumulation. Specifically, we postulated that certain fungal taxa may promote or constrain nucleoside biosynthesis, providing novel insights into which fungi contribute to the quality of *O. sinensis*. To test this hypothesis, Illumina high-throughput sequencing was employed to characterize the diversity and structure of mycosphere-associated fungal communities across multiple production regions. Simultaneously, nucleoside contents in the corresponding *O. sinensis* samples were quantified and compared. By integrating microbial and chemical data, this study aimed to reveal the potential relationship between mycosphere fungal profiles and nucleoside accumulation of *O. sinensis*. The results would deepen understanding of how mycosphere fungi influence nucleoside-based quality in *O. sinensis*.

## 2. Materials and Methods

### 2.1. Sample Collection

*O. sinensis* and its mycosphere soil samples were collected from eight different geographical regions: Songpan County in Aba Prefecture, Sichuan Province (AB); Litang County in Garze Prefecture, Sichuan Province (GZ); Jiuzhi County in Guoluo Prefecture, Qinghai Province (GL); Zhiduo County in Yushu Prefecture, Qinghai Province (YS); Maqu County in Gannan Prefecture, Gansu Province (MQ); Shangri-La in Diqing Prefecture, Yunnan Province (DQ); Lang County in Nyingchi Prefecture, Tibet Autonomous Region (LZ); and Dingqing County in Chamdo Prefecture, Tibet Autonomous Region (CD). Specific geographical information is provided in Table 1. Within each region, samples were collected from three independent plots separated by approximately 100 m. From each plot, at least 20 *O. sinensis* and corresponding soil samples were collected, and five of them were randomly selected and pooled as one group, defined as a biological replicate, resulting in three replicates per region.

All *O. sinensis* samples were collected as intact fungal–host complexes, including the mummified insect larva (sclerotium) and the fruiting body. During soil sampling, surface vegetation was removed, and soils were collected within a radius of 1–2 cm around *O. sinensis* at a depth of 5–10 cm. Following collection, samples were preserved at 4 °C, promptly transported to the laboratory, and subsequently stored at −80 °C until further analysis. In the laboratory, soil particles naturally adhering to the sclerotia were carefully brushed off and combined with the surrounding soil samples. The mixed soil was then sieved through a 2 mm mesh to remove plant residues and stones, yielding the final soil samples used for downstream analyses.

### 2.2. Sample DNA Extraction and PCR Amplification

Gravel, biofilm, and other debris were carefully removed from the mycosphere soil samples, followed by sieving. Genomic DNA was extracted from the sieved soil samples using the MagBeads FastDNA Kit for Soil. The quality and integrity of the extracted DNA were verified by 0.8% agarose gel electrophoresis, and DNA concentration and purity were measured using a NanoDrop spectrophotometer. The fungal ITS V1 region was amplified using the specific primers ITS5 (5′—GGAAGTAAAAGTCGTAACAAGG—3′) and ITS2 (5′—GCTGCGTTCTTCATCGATGC—3′). Each 25 μL PCR mixture contained 0.25 μL ABclonal DNA polymerase, 5 μL 5 × Reaction Buffer, 5 μL 5 × High GC Buffer, 2 μL dNTPs (10 mM), 2 μL template DNA, 1 μL forward primer (10 μM), 1 μL reverse primer (10 μM), and 8.75 μL nuclease-free water. PCR amplification was performed on a thermal cycler under the following conditions: initial denaturation at 98 °C for 5 min; followed by 30 cycles of denaturation at 98 °C for 30 s, annealing at 55 °C for 30 s, and extension at 72 °C for 45 s; with a final extension at 72 °C for 5 min. PCR products were verified by 2% agarose gel electrophoresis. Target fragments were excised and purified using a magnetic bead-based separation method.

### 2.3. High Throughput Sequencing

Prior to sequencing, the quality of the constructed libraries was assessed using the Agilent 2100 Bioanalyzer (Agilent Technologies, Santa Clara, CA, USA) in conjunction with the Agilent High Sensitivity DNA Kit to ensure appropriate fragment size distribution and integrity. Library concentration was then quantified using the Quant-iT™ PicoGreen^®^ dsDNA Assay Kit (Thermo Fisher Scientific, Waltham, MA, USA) on a Promega QuantiFluor^®^ fluorometer, following the manufacturer’s instructions. Only libraries that met the quality control criteria were retained for sequencing. To ensure accuracy during multiplexed sequencing, all index sequences were unique and non-redundant. Qualified libraries were diluted to the required concentrations, pooled in equimolar ratios, and denatured using NaOH to generate single-stranded DNA templates suitable for cluster generation. Sequencing was performed on the Illumina NovaSeq 6000 platform (Illumina, San Diego, CA, USA) using a paired-end 250 bp (PE250) strategy, according to the standard protocols provided by the sequencing service provider.

### 2.4. Nucleoside Content Determination

The *O. sinensis* samples were subjected to freeze-drying, followed by grinding into a fine powder. A sample mass of 40 mg was then combined with 1.6 mL of double-distilled water and subjected to ultrasonic extraction at 300 W and 50 °C for a duration of 40 min. Subsequently, the mixture was centrifuged at 10,000 rpm for 10 min, and the resulting supernatant was filtered using a 0.45 μm filter membrane. Chromatographic analysis was conducted under the following conditions: the chromatographic column utilized was a Hypersil BDS C_18_ (4.6 mm × 250 mm, 5 μm). The mobile phase consisted of methanol (A) and 0.3% acetic acid in water (B). The flow rate was maintained at 1 mL/min, and the injection volume was set at 20 μL. Detection was performed at a wavelength of 260 nm using an Agilent LC 1260 high-performance liquid chromatograph (Agilent Technologies, Santa Clara, CA, USA). The elution gradient was programmed as follows: 0 to 10 min, 100% B; 10 to 20 min, 100% to 92% B; 20 to 30 min, 92% to 80% B; and 30 to 40 min, 80% B. Standard solutions were prepared by dissolving adenosine, inosine, guanosine, cytidine, and uridine standards to achieve solutions with concentrations of 0.5 μg/mL, 1 μg/mL, 5 μg/mL, 10 μg/mL, 25 μg/mL, 50 μg/mL, 75 μg/mL, and 100 μg/mL. These standard solutions were subjected to HPLC under the same chromatographic conditions described above. Peak areas were used to establish calibration curves for quantitative analysis.

### 2.5. Data Analysis

Raw sequencing data were processed in QIIME2 (version 2019.4) with the DADA2 plugin for quality control, denoising, merging, and chimera removal (Table S1). Taxonomic assignment of amplicon sequence variants (ASVs) was performed using the UNITE database, and abundance tables were generated across multiple taxonomic levels. Sequencing depth analysis was performed using R (version 4.4.1) by randomly subsampling the total sequence counts for each sample in the ASV abundance matrix at different depths. Rarefaction curve and rank-abundance plots were generated based on the number of sequences obtained at each depth and the corresponding ASV counts to assess community diversity and evenness. Microbial community compositions were visualized with box plots, alpha diversity indices were tested using the Shapiro-Wilk test and the Kruskal-Wallis test, followed by post hoc comparisons with the Conover-Dunn test. Beta diversity was calculated using the Jaccard distance metric in R (version 4.1.1) with the vegan package. Group differences in community composition were statistically evaluated using permutational multivariate analysis of variance (PERMANOVA) with 999 permutations. The resulting distance matrix was used to investigate variations in microbial community structure. Differentially enriched taxa were identified using linear discriminant analysis effect size (LEfSe). Microbial co-occurrence networks were constructed using SparCC, with correlation coefficients and significance determined using the RMThreshold package in R version 4.4.1. Nucleoside contents and relative abundances at the phylum and genus levels were tested using analysis of variance (ANOVA), and statistical analyses were performed with IBM SPSS Statistics (version 27). All data visualization was conducted in Origin (version 2022) and R (version 4.4.1). Pearson correlation analysis was used to evaluate linear associations among the mycosphere fungal communities and nucleoside content, with statistical significance set at *p* < 0.05.

## 3. Results

### 3.1. Diversity of Mycosphere Fungal Communities Associated with O. sinensis

A total of 3,007,985 high-quality sequences were generated from 24 samples during this sequencing process, resulting in the identification of 2276 ASVs. Rarefaction curves approached plateau (Figure S1A), reached a plateau at approximately 10,000 reads for most samples, indicating sufficient sequencing depth to capture the majority of fungal diversity. The Rank abundance curve was generally steep, with a sharp decline at the beginning (Figure S1B), indicating that the dominant species dominated in the community, and the abundance of non-dominant species was significantly low. By analyzing the normalized ASV data, the microbial community composition across taxonomic levels was profiled for each sample, allowing direct comparison of taxonomic richness among different samples. As shown in Figure S1C, samples from YS and GL contained a higher number of ASVs at all taxonomic levels, indicating richer community structures in these regions.

**Figure 1:**
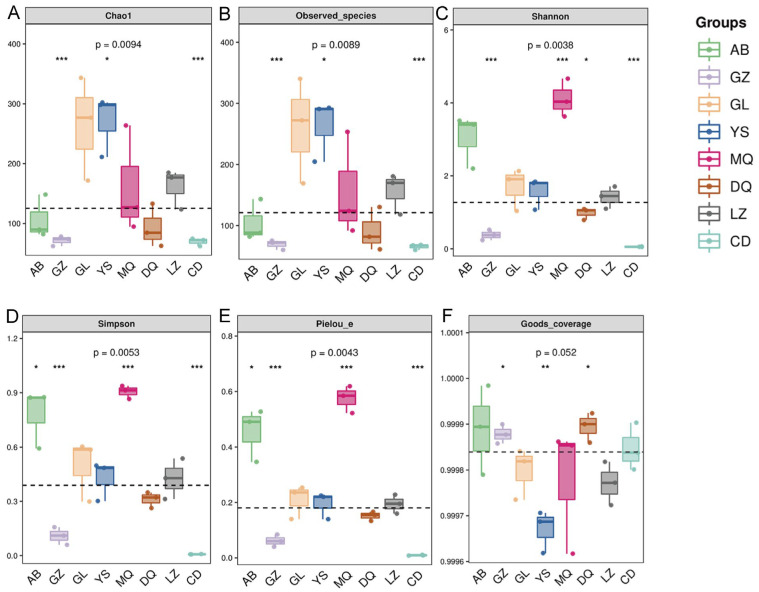
Alpha diversity indices of fungal communities in the mycosphere soil of *O. sinensis* from different geographical regions. (**A**) Chao1 index; (**B**) Observed species index; (**C**) Shannon index; (**D**) Simpson index; (**E**) Pielou species evenness index; (**F**) Good’s coverage (* *p* < 0.05, ** *p* < 0.01, *** *p* < 0.001).

Alpha diversity indices were used to assess species richness, diversity, and evenness within each sample. Chao1 and Observed species indices were higher in GL and YS samples compared with those in others, indicating the richer fungal communities (>250) (Figure 1A,B). These were followed by MQ and LZ (>150), while AB and DQ had moderate richness (>90), and GZ and CD exhibited the lowest richness (>60, *p* < 0.001). The Shannon index and Simpson index were used to measure species diversity, and the Pielou index was used to measure species evenness. The results showed that MQ and AB had higher fungal species diversity and evenness, followed by GL, YS, DQ, and LZ, while GZ and CD had the lowest values (*p* < 0.001) (Figure 1C–E). The Good’s coverage index showed no significant differences among samples (*p* > 0.05), and all samples had coverage values exceeding 97%, indicating that the sequencing depth was sufficient to support subsequent community structure analyses (Figure 1F).

**Figure 2:**
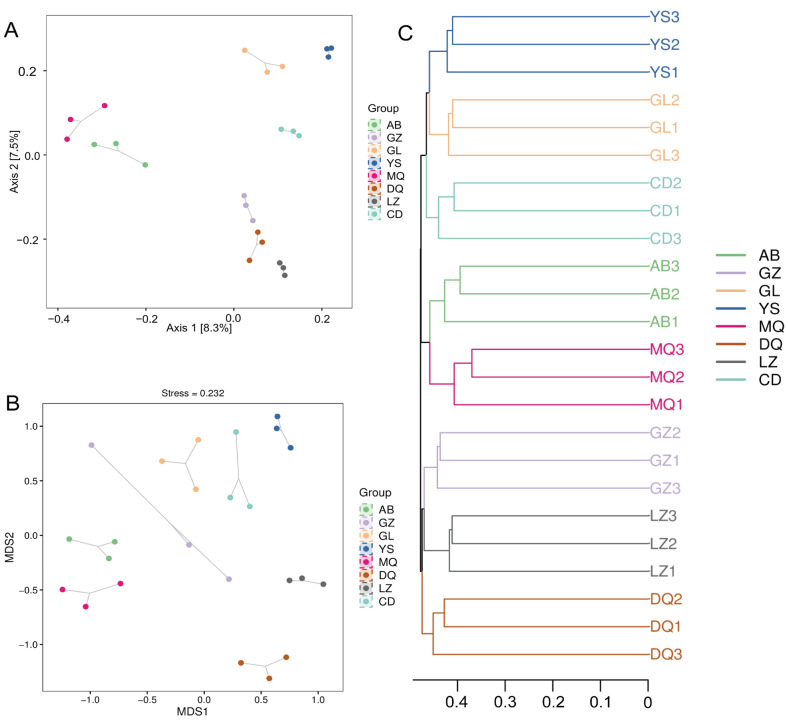
Beta diversity analysis of fungal communities in the mycosphere soil of *O. sinensis* from different geographical regions. (**A**) PCoA analysis; (**B**) NMDS analysis; (**C**) UPGMA analysis.

To explore the similarity of peridial soil fungal communities of *O. sinensis* from different regions, beta diversity analysis was performed using principal coordinates analysis (PCoA), non-metric multidimensional scaling analysis (NMDS), and unweighted pair group method with arithmetic mean (UPGMA). The PCoA results demonstrated that beta diversity analysis indicated significant differences in fungal community structure across various regions (*R* = 0.861, *p* = 0.001). Consistently, PERMANOVA further confirmed that the mycosphere fungal communities of *O. sinensis* varied significantly among regions (*R*^2^ = 0.44, *p* = 0.001). Samples from MQ and AB producing areas are closer (Figure 2A), indicating similar community composition of these samples. YS, GL, and CD also grouped, suggesting compositional similarity among them. In contrast, samples from GZ and LZ clustered near those from DQ, indicating another group of shared community traits. The results of NMDS and UPGMA were consistent with the PCoA results, confirming the observed clustering patterns among production regions (Figure 2B,C).

### 3.2. Fungal Community Composition in the Mycosphere Soil of O. sinensis

**Figure 3:**
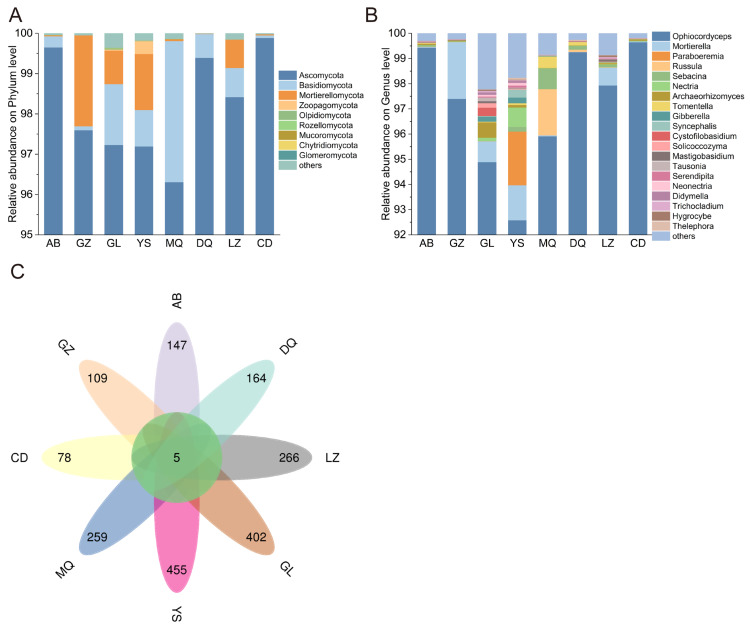
(**A**) Distribution of the top 10 fungal phyla in the mycosphere soil of *O. sinensis* across different geographical regions. (**B**) Distribution of the top 20 fungal genera in the mycosphere soil of *O. sinensis* across different geographical regions. (**C**) ASVs distribution of mycosphere soil fungi of *O. sinensis* from different geographical regions.

Fungi identified in all soil samples included 12 phyla, 35 classes, 83 orders, 188 families, and 343 genera. At the phylum level, Ascomycota absolutely dominated in all samples across eight different geographical regions, with a relative abundance ranging from 96.30% to 99.88%. Besides, Basidiomycota and Mortierellomycota were also observed at higher relative abundances. Among them, Basidiomycota showed the highest abundance in AB, while Mortierellomycota was most abundant in GZ (0.23%). Zoopagomycota only showed higher relative abundance in YS compared to those in others (0.31%) (Figure 3A).

At the genus level, *Ophiocordyceps* was predominant in all soil samples, accounting for 92.58% (YS) to 99.65% (CD) of the total fungal community. In addition to *Ophiocordyceps*, *Mortierella* was present across multiple sites, although its abundance was consistently lower than that of *Ophiocordyceps*. The highest abundance of *Mortierella* was observed in GZ (2.25%), while the lowest was in CD (0.25%). Comparative analysis of the genus-level composition revealed higher fungal diversity in the GL and YS regions. In GL, genera with a relative abundance greater than 0.3% included *Ophiocordyceps*, *Mortierella*, *Archaeorhizomyces*, and *Cystofilobasidium*. In YS, the dominant genera (relative abundance > 0.3%) were *Ophiocordyceps*, *Mortierella*, *Paraboeremia*, *Nectria*, and *Syncephalis*. Certain genera exhibited regional specificity in their abundance. For example, *Russula* showed the highest abundance exclusively in MQ (1.84%); *Sebacina* peaked in DQ (0.15%), and *Archaeorhizomyces* had the highest abundance in CD (0.52%) (Figure 3B).

### 3.3. Comparative Analysis of Mycosphere Fungal Communities Associated with O. sinensis

To analyze the differences in mycosphere soil fungal communities of *O. sinensis*, petal diagrams were drawn based on the total identified ASVs. As shown in Figure 3C, five ASVs were shared across all groups, while each region harbored distinct sets of unique ASVs, including 147 in AB, 109 in GZ, 455 in YS, 402 in GL, 259 in MQ, 164 in DQ, 266 in LZ, and 78 in CD. Based on the ASV abundance at various taxonomic levels, bar plots were constructed to show the top 10 phyla and genera with the highest abundance. The results revealed that all shared ASVs among the eight sample groups belonged to Ascomycota and *Ophiocordyceps*.

To further investigate community composition differences, a hierarchical clustering heatmap was constructed using the top 50 genera based on average relative abundance. As shown in Figure S2, red blocks indicated genera with higher relative abundance in given samples, while blue blocks represented lower abundance. Among all groups, GL exhibited the greatest number of highly abundant genera (26 in total), indicating a more complex fungal community structure. These genera, clustered in Class II, included *Alternaria*, *Clavaria*, *Tausonia*, *Preussia*, *Schizothecium*, *Didymella*, *Cladophialophora*, *Acicuseptoria*, *Lyonectria*, *Hygrocybe*, *Mastigobasidium*, *Trichocladium*, *Rachicladosporium*, *Gibberella*, *Neonectria*, and *Humicola*. YS was characterized by 13 genera with higher relative abundance, clustered in Class III, including *Thelephora*, *Serendipita*, *Naganishia*, *Rachicladosporium*, *Gibberella*, *Neonectria*, *Humicola*, etc. Notably, *Ophiocordyceps* had the lowest relative abundance in YS compared to other regions. In addition, LZ had a relatively high abundance of certain genera, which were clustered into Class I, such as *Cortinarius*, *Phialocephala*, *Inocybe*, and *Pseudeurotium*. In MQ, *Russula*, *Hygrophorus*, *Tomentellopsis*, *Sebacina*, and *Tomentella* were more abundant and clustered into Class IV. For AB, GZ, and CD, only a single dominant genus was observed in each region, namely *Thermomyces* (AB), *Mortierella* (GZ), and *Millerozyma* (CD), respectively.

**Figure 4:**
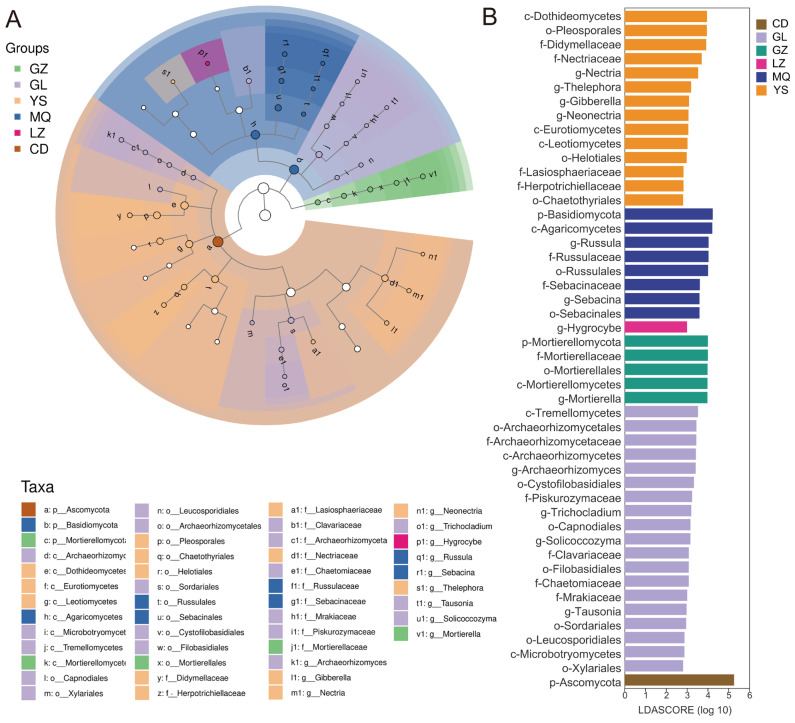
Biomarkers in different samples were identified through LEfSe analysis. (**A**) Linear discriminant analysis (LDA) of mycosphere soil fungi associated with *O. sinensis* from different geographical regions. (**B**) Comparisons of the different groups of soil fungi with LDA score > 3, *p* < 0.05.

To identify potential microbial biomarkers, linear discriminant analysis effect size (LEfSe) was conducted to detect significantly different taxonomic units among samples. The threshold was set at LDA score > 2 and *p* < 0.01 for statistical significance. Figure 4A illustrates the taxonomic tree at different levels. LEfSe analysis identified a total of 48 significantly different taxa, with six major groups of potential biomarkers across the eight sample regions. Specifically, GL had 19 distinct biomarkers, including Archaeorhizomycetes, Archaeorhizomycetales, Archaeorhizomycetaceae, *Archaeorhizomyces*, Tremellomycetes, and Cystofilobasidiales. YS had 14 biomarkers, such as Dothideomycetes, Eurotiomycetes, Leotiomycetes, etc. MQ showed 8 key taxa, including Basidiomycota, Agaricomycetes, and Russulales. GZ had 5 key markers, including Mortierellomycetes, Mortierellales, Mortierellaceae, and *Mortierella*. Only one key biomarker was detected in LZ (*Hygrocybe*) and CD (Ascomycota). Overall, the majority of biomarker taxa belonged to the phyla Ascomycota, Basidiomycota, and Mortierellomycota, which was consistent with previous findings from diversity analysis and community composition comparisons (Figure 4B).

### 3.4. Network Analysis of Mycosphere Fungal Communities Associated with O. sinensis

**Figure 5:**
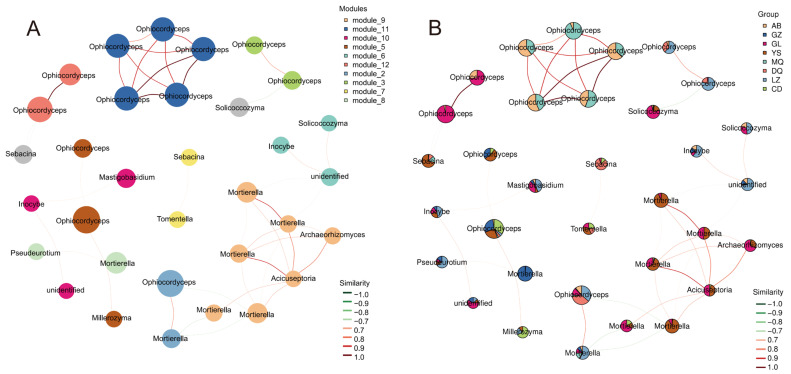
(**A**) Modular advantage seed network diagram. (**B**) Dominant seed network diagram with grouped abundance pie chart. The red line indicates a positive correlation, and the green line indicates a negative correlation.

To identify the dominant fungal taxa in the mycosphere networks of *O. sinensis* across different regions, only ASVs with a total sequence count ≥ 10 and present in at least five samples were retained. Based on these filtered ASVs, a correlation matrix was constructed using the SparCC algorithm to reveal potential co-occurrence patterns among fungal taxa, based on *R* > 0.7 and *p* < 0.05. At the phylum level, the network consisted of three major fungal phyla: Ascomycota, Basidiomycota, and Mortierellomycota (Figure S3). These phyla were distributed across 10 network modules (Figure 5A). *Ophiocordyceps* was exclusively distributed in Module 11 and Module 12. Module 11 comprised samples from MQ and AB, while Module 12 included samples from GL and AB. A strong positive correlation was observed between the node degrees of these two modules. In addition, *Ophiocordyceps* in Module 12 showed negative correlations with *Sebacina*, involving samples from YS, MQ, and DQ. Module 3 was composed of *Ophiocordyceps*, which showed positive correlations in samples from LZ and DQ, and negative correlations with *Solicoccozyma* in samples from GL and YS. Module 5 contained both *Ophiocordyceps* and *Mortierella*, spanning all sample groups. Module 9 included *Mortierella*, *Archaeorhizomyces*, and *Acicuseptoria*, and was primarily associated with samples from YS, GL, and CD. A negative correlation was detected between Module 9 and Module 2, where Module 2 encompassed samples from all regions and was composed of *Ophiocordyceps* and *Mortierella* (Figure 5B). In summary, based on its high degree and high betweenness, *Ophiocordyceps* emerged as the core taxon in the mycosphere fungal communities of *O. sinensis* across all regions, playing a central and potentially driving role in shaping the structure of the fungal networks.

### 3.5. Analysis of Nucleoside Content in O. sinensis from Different Geographical Regions

**Figure 6:**
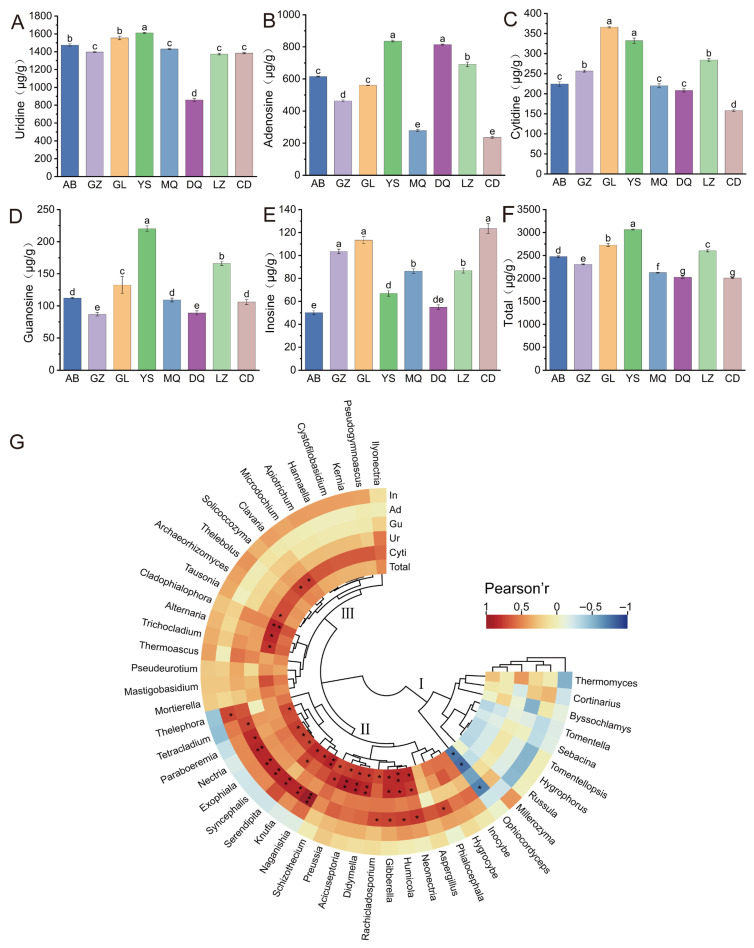
(**A**) Uridine, (**B**) adenosine, (**C**) cytidine, (**D**) guanosine, (**E**) inosine, (**F**) total nucleoside content; (**G**) correlation analysis between the top 50 most abundant fungal genera in the mycosphere soil of *O. sinensis* from different geographical regions and the nucleoside content of *O. sinensis* (* *p* < 0.05, ** *p* < 0.01, *** *p* < 0.001). Different lowercase letters indicate significant differences.

As shown in Figure 6A–F, the contents of the five major nucleosides in *O. sinensis* samples from different geographic regions varied considerably. Among them, uridine (859.0–1611.0 μg/g), adenosine (235.4–835.6 μg/g), and cytidine (157.9–365.9 μg/g) were present at relatively higher concentrations, whereas guanosine (86.8–220.3 μg/g) and inosine (50.1–123.4 μg/g) showed lower levels. The total nucleoside content ranged from 2006.9 μg/g to 3066.0 μg/g across samples, with the descending order being: YS > GL > LZ > AB > GZ > MQ > DQ > CD. Variance analysis revealed that the total nucleoside contents in YS, GL, and LZ were significantly higher than those in other regions (*p* < 0.05), whereas no significant difference was observed between DQ and CD (*p* > 0.05). Further comparative analysis of individual nucleosides demonstrated distinct regional patterns. Adenosine levels were higher in YS and DQ compared to other samples. Cytidine contents were relatively elevated in GL and YS. Additionally, guanosine contents were significantly higher in YS and LZ than in other areas (*p* < 0.05). For inosine, samples from GZ, GL, and CD exhibited comparatively higher levels, but no significant differences were observed among them (*p* > 0.05). Notably, uridine content in YS was the highest and differed significantly from other regions (*p* < 0.05), whereas DQ had the lowest level. These results collectively underscored the geographic influence on the accumulation patterns of nucleosides in *O. sinensis*, with certain regions showing pronounced enrichment of specific nucleoside types.

### 3.6. Correlation Analysis Between Abundance of Mycosphere Soil Fungal Communities and Nucleoside Content in O. sinensis

To explore the potential relationships between the mycosphere fungal communities and nucleoside content in *O. sinensis* across different regions, correlation analysis was conducted between the top 50 most abundant fungal genera and the contents of five nucleosides in *O. sinensis* samples. Based on the correlation patterns between fungal genera and nucleoside content, the analyzed genera were grouped into three distinct clusters (Figure 6G), reflecting differences in the direction and magnitude of their associations with specific nucleosides. In Cluster I, *Ophiocordyceps* exhibited a significant negative correlation with cytidine, guanosine, and total nucleoside content (*R* > −0.8, *p* < 0.05). Cluster II included a group of genera such as *Neonectria*, *Humicola*, *Gibberella*, *Didymella*, *Acicuseptoria*, *Nectria*, and *Serendipita* and others, which exhibited strong positive correlations with total nucleoside content, as well as cytidine, guanosine, and adenosine (*R* > 0.7, *p* < 0.05). In Cluster III, a positive correlation was observed primarily with cytidine (*R* > 0.7, *p* < 0.05), involving genera such as *Trichocladium*, *Alternaria*, *Cladophialophora*, *Tausonia*, *Solicoccozyma*, and *Clavaria*. Overall, the total nucleoside content in *O. sinensis* exhibited positive associations with a range of other fungal genera, such as *Humicola*, *Naganishia*, *Didymella*, *Gibberella*, and others. These findings highlighted a potential link between the mycosphere fungal community composition and the biosynthesis or accumulation of nucleoside components in *O. sinensis*.

## 4. Discussion

*O. sinensis* and its surrounding mycosphere soils are inhabited by diverse and abundant fungal communities, which differ significantly across cultivation modes, tissue compartments, and geographic regions [3,19]. These community variations may be closely related to the host infection, stroma development, and occurrence of *O. sinensis*, and ultimately the characteristics of its medicinal quality. In addition, soil fungi represent indispensable components of fragile alpine ecosystems in the Qinghai-Tibet Plateau by participating in critical biogeochemical processes, including organic matter degradation, nutrient recycling, and carbon turnover [6]. Thus, characterizing the fungal community composition of natural *O. sinensis* is of great importance for clarifying the mechanisms regulating the occurrence, quality formation of this valuable medicinal fungus.

In the present study, mycosphere soil fungal communities from different regions were predominantly composed of three phyla, including Ascomycota, Basidiomycota, and Mortierellomycota (Figure 3A), indicating that the main fungal groups in the mycosphere soils of *O. sinensis* were stable across different production regions. These findings were consistent with previous reports [9,20]. Members of the Ascomycota fungi are commonly saprophytes, which play important roles in carbon and nitrogen cycling in grassland ecosystems by decomposing plant residues and degrading soil organic matter [21]. Basidiomycota fungi are characterized by their strong ability to decompose complex organic carbon compounds such as lignocellulose and cellulose [22]. Furthermore, Mortierellomycota fungi produce oxalic acid that facilitates the solubilization of inorganic phosphates, thereby influencing soil pH and nutrient availability [23]. The dominance of Ascomycota, Basidiomycota, and Mortierellomycota is therefore likely to sustain organic matter decomposition and nutrient cycling in fragile high-altitude habitats, ultimately providing a stable nutritional foundation for the growth and reproduction of *O. sinensis*.

At the genus level, the genus *Ophiocordyceps* (represented by *O. sinensis*) was overwhelmingly dominant across all sampled mycosphere soils (Figure 3B). This suggested that *O. sinensis* mycelia substantially might contribute to the fungal community structure in its immediate soil microenvironment. Similar patterns have been reported previously, in which *Ophiocordyceps* consistently dominated the fungal communities of fruiting bodies, external mycelial cortices, and habitat soils of Chinese *Cordyceps* [8,24]. In addition, various fungi have been isolated and identified from natural *O. sinensis* and its habitat microenvironment, such as *Paecilomyces hepiali, P. sinensis, Mortierella hepiali, Beauveria bassiana*, and *Tolypocladium sinense* [25,26]. Notably, most of these fungi are entomopathogenic, capable of infecting *Thitarodes* larvae (the insect hosts of *O. sinensis*) and competing with *O. sinensis* for resources [27,28]. Thus, we propose that the enrichment of *Ophiocordyceps* populations in mycosphere soils might limit the colonization of other insect-pathogenic fungi through nutrient competition or host exclusion, which would ultimately benefit the infection process and occurrence of *O. sinensis*.

The α-diversity analysis revealed that fungal community richness and diversity in the mycosphere soils from YS and GZ differed significantly from those of the other six production areas (Figure 1A–E). However, β-diversity analysis indicated that despite some variations in community composition across samples (Figure 2A–C). Together, these results suggested that while the within-sample fungal diversity (α-diversity) was relatively high and variable among regions, the overall community composition between regions (β-diversity) remained relatively conserved. This finding aligned with previous studies reporting rich intraspecific diversity but limited interregional differences in fungal communities associated with *O. sinensis* [24]. Previous studies have shown that altitude and aspect on the Qinghai-Tibet Plateau significantly influence the composition and diversity of fungal communities [29]. Thus, the alterations in fungal community structures across different regions may be influenced by factors such as climate, vegetation, soil heterogeneity, and vertical stratification. However, the specific interactions among these factors remain to be further elucidated.

Furthermore, network analysis highlighted that Ascomycota, particularly *Ophiocordyceps*, constituted a keystone taxon within the mycosphere fungal network, exerting a driving influence on community structure and stability (Figure 5A,B). LEfSe analysis further revealed that each production area harbored its characteristic fungal taxa (Figure 4A,B), which may contribute to the observed quality differences among regions. In particular, the LDA bar plot (Figure 4B) showed that *Hygrocybe* was significantly enriched exclusively in LZ samples. Previous studies have demonstrated that *Hygrocybe* is sensitive to inorganic nitrogen and occurs predominantly in nitrogen-deficient habitats [30], while Lodge et al. [31] reported that *Hygrocybe* exhibits strong tolerance to the harsher climatic conditions of grassland habitats, characterized by pronounced diurnal and seasonal fluctuations in temperature and humidity. These findings suggest that the soil environment of LZ *O. sinensis* production areas may be more challenging than that of other regions. Another noteworthy observation from our differential abundance analysis was that fungal taxa significantly enriched in GZ samples were consistently assigned to Mortierellomycota, spanning from the phylum to the genus level. Mortierellomycota are typically abundant in soils rich in organic matter, where they can influence soil microbial communities by altering nutrient acquisition and microhabitats, thereby indirectly affecting nutrient cycling and availability [32]. Moreover, members of this phylum are capable of releasing organic acids to solubilize phosphorus in soils [33]. These findings suggest that the soil environment of GZ *O. sinensis* production areas may be more nutrient-rich compared with other regions. Collectively, the distinct fungal taxa enriched in the mycosphere soils across different production areas may reflect adaptations to their respective edaphic conditions, warranting further investigation.

Nucleosides are key chemical markers for evaluating the quality of *O. sinensis*, underpinning its core pharmacological effects such as anti-inflammatory, anti-tumor, and organ-protective activities, as well as serving as important indicators for authenticity verification and assessment of cultivated substitutes [34]. Our results showed significant variation in nucleoside content among *O. sinensis* across different production areas. Notably, total nucleoside, adenosine, uridine, and guanosine contents were highest in YS samples, consistent with prior studies [35]. It was worth mentioning that the fungal genus abundance and diversity at the phylum level were highest in the mycosphere soils of *O. sinensis* from GL and YS, which also exhibited relatively higher nucleoside contents. This may be attributed to their shared geographical location within the Sanjiangyuan region, characterized by higher altitude and abundant precipitation (annual average rainfall ranging from 262.2 to 772.8 mm), resulting in soil types more conducive to fungal growth [24,36]. However, these findings differed somewhat from previous reports, possibly due to variations in sample collection, processing methods, technical approaches, or local microenvironmental heterogeneity. The elevated fungal diversity observed in GL and YS soils may contribute to a more stable and resilient ecosystem that enhances the stress tolerance and quality of *O. sinensis* [37]. Compared to other sampling sites, these regions were likely to support richer vegetation and more complex ecosystem structures, offering diverse ecological niches for fungal communities.

Several of the 50 most abundant fungal genera exhibited significant correlations with nucleoside contents, including *Naganishia*, *Acicuseptoria*, *Nectria*, *Serendipita*, *Humicola*, and *Ophiocordyceps*. *Naganishia* is a psychrophilic genus typically restricted to habitats with persistently low temperatures or cold climates [38], and its xylanase activity, coupled with nutritional competition with other pathogens, creates a favorable microenvironment that has been shown to promote tomato seedling growth [39]. *Serendipita* has been extensively studied as a root-colonizing fungus that forms symbiotic associations with more than 200 plant species, enhancing plant growth and improving nutrient and water uptake [40], as well as conferring tolerance against various biotic and abiotic stresses [41]. *Humicola* is a genus of saprophytic fungi involved in carbon and nitrogen cycling. Duan et al. [42] reported that members of this genus are sensitive to exogenous nitrogen inputs and play an important role in soil carbon sequestration. Moreover, Stanek [43] demonstrated that *Humicola* can produce vitamins and amino acids that can be utilized by *Aspergillus bisporus* for growth. *Nectria* is recognized as a dominant endophytic fungus colonizing ginseng roots [44], whose metabolites present quinones and phenols as chemical constituents and exhibit antibacterial activity against bacterial pathogens such as *Staphylococcus aureus*, *Enterococcus faecalis*, and *Phytophthora avellaneum* [45], and it has also been reported to improve tomato growth [46]. In the study, *Naganishia*, *Acicuseptoria*, *Nectria*, *Serendipita*, and *Humicola* showed significant positive correlations with nucleoside contents (Figure 6G). These observations suggest that such fungi, through their metabolic activities and interactions with other microbial communities in the mycosphere, might play a contributory role in the biosynthesis or accumulation of nucleosides in *O. sinensis*, potentially influencing its bioactive compound profile and overall medicinal quality. *Acicuseptoria* has been described by Quaedvlieg et al. [47] as a segregate of the polyphyletic genus *Septoria*, characterized by brown, globose pycnidia, and in the study, it was positively correlated with nucleoside contents in *O. sinensis*, although its specific functional role requires further investigation. Notably, *Ophiocordyceps* (represented by *O. sinensis*) exhibited a significant negative correlation with nucleoside contents (Figure 6G). This phenomenon may be explained by nutritional competition, as the mycelial form of *O. sinensis* in mycosphere soil and the fruiting body–larva complex represent different developmental stages of the same species and share similar carbon and nitrogen demands [48].

In addition to altitude, ecological covariates such as vegetation and soil type likely influence *O. sinensis* growth by jointly shaping the microclimate, organic matter input, and physicochemical properties of the mycosphere soil. These factors modulate fungal diversity, metabolic activity, and interactions (both symbiotic and competitive), which in turn co-vary with nucleoside accumulation. While the present study provides a preliminary exploration of correlations between mycosphere fungal communities and nucleoside contents, causality remains unresolved, and the dominance of *Ophiocordyceps* may obscure signals from low-abundance taxa. To further disentangle the relative contributions of environmental and biotic factors, future studies should integrate high-resolution ecological metadata, including altitude, vegetation characteristics, and soil physicochemical parameters such as pH, texture, moisture content, and organic matter. Moreover, targeted analyses of rare taxa will be necessary to evaluate whether low-abundance fungi disproportionately explain variations in nucleoside levels. Combining such ecological information with mechanistic approaches, including metagenomics, transcriptomics, metabolomics, and controlled experimental assays, will be essential to reveal how soil microbial communities and environmental factors jointly regulate nucleoside biosynthesis in *O. sinensis*.

## 5. Conclusions

This study systematically characterized the composition and regional variation of mycosphere fungal communities associated with *O. sinensis* across major production areas in the Qinghai-Tibetan Plateau, and explored their potential links with nucleoside accumulation. High-throughput sequencing revealed a consistently dominant presence of Ascomycota, Basidiomycota, and Mortierellomycota across all regions, with *Ophiocordyceps* being the most abundant genus. Despite this stability at higher taxonomic levels, significant regional differences were observed in community composition, diversity, and indicator taxa. Importantly, these regional differentiation patterns of fungal communities corresponded with marked variation in nucleoside contents, particularly in samples from Yushu (YS) and Guoluo (GL), which exhibited both higher fungal diversity and elevated levels of adenosine, uridine, and guanosine. Correlation analysis further revealed that the abundance of multiple fungal taxa in the mycosphere soil of *O. sinensis* was positively linked with nucleoside accumulation, such as *Naganishia, Acicuseptoria, Nectria, Serendipita, and Humicola*. Collectively, these results indicate that the composition of mycosphere fungal communities is closely associated with nucleoside accumulation in *O. sinensis*, supporting the hypothesis that the microbiome contributes to its medicinal quality. While causality requires experimental validation, the correlations observed suggest that specific fungal taxa may influence nucleoside biosynthesis through metabolic, symbiotic, or competitive interactions. Importantly, this highlights the potential to manipulate the mycosphere through ecological regulation of soil fungal diversity and activity as a strategy to enhance metabolite accumulation and improve ecological cultivation of this valuable fungus.

## Figures and Tables

**Table 1:** Geographic Information of Samples.

Sample Number	Collecting Locations	Longitude/E	Latitude/N	Elevation/m
AB	Songpan County, Aba Prefecture, Sichuan Province	102°13′16″	31°12′28″	3230
GZ	Litang County, Ganzi Prefecture, Sichuan Province	101°57′39″	30°3′7″	3150
GL	Jiuzhi County, Guoluo Prefecture, Qinghai Province	100°26′45″	34°31′50″	4340
YS	Zhiduo County, Yushu Prefecture, Qinghai Province	96°55′57″	33°01′56″	4498
MQ	Maqu County, Gannan Prefecture, Gansu Province	102°11′48″	34°3′16″	3980
DQ	Shangri-la, Diqing Prefecture, Yunnan Province	99°42′43″	28°33′54″	4260
LZ	Lang County, Nyingchi Prefecture, Tibet Autonomous Region	93°15′12″	29°54′49″	4122
CD	Dingqing County, Chamdo Prefecture, Tibet Autonomous Region	97°13′44″	33°23′27″	4244

## Data Availability

The original contributions presented in this study are included in the article/Supplementary Materials. Further inquiries can be directed to the corresponding author(s).

## Supplementary Materials

The following supporting information can be downloaded at: https://www.mdpi.com/article/10.3390/jof11100696/s1. Figure S1: (A) Rarefaction curves, (B) Rank abundance curves, (C) the number of microbial taxonomic units at each classification level. Figure S2. The heatmap of species composition of the top 50 genera level biclustering of *O. sinensis* abundance in different habitats. Figure S3: Network diagram of fungal taxa at the phylum level. The red line indicates a positive correlation, and the green line indicates a negative correlation. Table S1. Per-sample statistics of sequencing reads before and after quality filtering and denoising.
