# Supplementary material for: Insights into the Mycosphere Fungal Community and Its Association with Nucleoside Accumulation in Ophiocordyceps sinensis
Source: J Fungi (Basel). 2025 Sep 25;11(10):696. doi: 10.3390/jof11100696 (PMC12565341; doi:10.3390/jof11100696)
Supplement: Supplementary file 1 [file jof-11-00696-s001.zip › supplementary materials.pdf]

Table S1. Per-sample statistics of sequencing reads before and after quality filtering and denoising.

SampleID	Input	Filtered	Denoised	Merged	Non-chimeric	Non-singleton
AB1	87390	82343	82131	82058	81387	81387
AB2	114850	108300	108084	107915	106551	106551
AB3	109033	102543	102346	102301	101232	101232
GZ1	114020	107898	107723	107635	107593	107593
GZ2	103537	97719	97568	97525	96625	96625
GZ3	116418	110065	109850	109800	109766	109766
GL1	111432	104795	104481	104287	104206	104206
GL2	110634	104481	104202	104109	103995	103995
GL3	107344	100659	100342	100105	100024	100024
YS1	123456	116309	116013	115884	115281	115281
YS2	132807	124617	124130	123819	123331	123331
YS3	122370	115282	114884	114632	113830	113830
MQ1	134555	128073	127757	127569	123276	123276
MQ2	125106	119034	118803	118737	117318	117318
MQ3	139699	132325	132117	131944	126152	126152
DQ1	116039	109861	109637	109554	109423	109423
DQ2	114884	108648	108493	108463	108003	108003
DQ3	109900	103889	103706	103689	103472	103472
LZ1	124730	117786	117495	117338	117248	117248
LZ2	125573	118505	118219	118105	117986	117986
LZ3	124282	117283	117060	116943	116862	116862
CD1	132308	124677	124491	124441	124441	124441
CD2	119276	112628	112448	112279	112264	112264
CD3	126101	119156	119000	118938	118916	118916

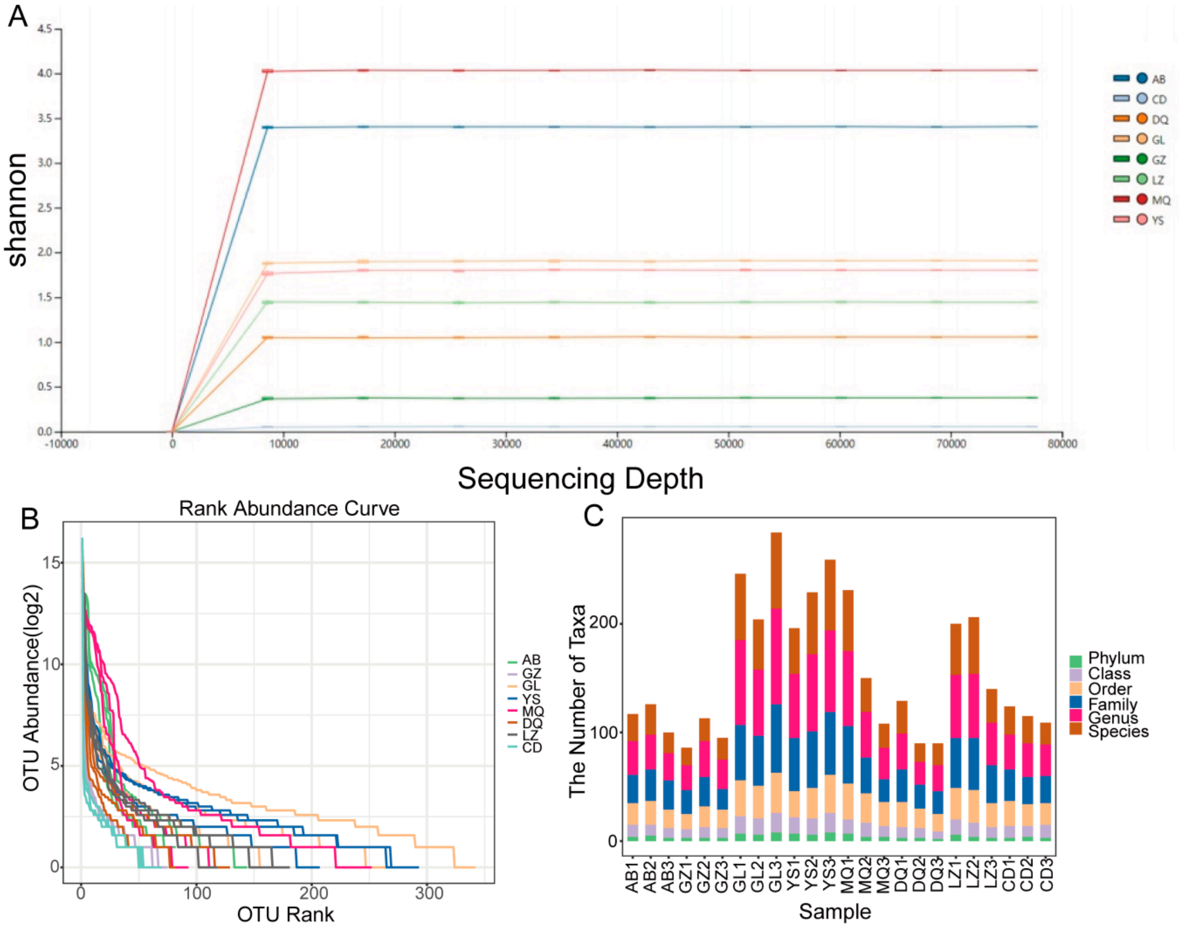

Figure S1. (A) Rarefaction curves, (B) Rank abundance curves, (C) the number of microbial taxonomic units at each classification level.

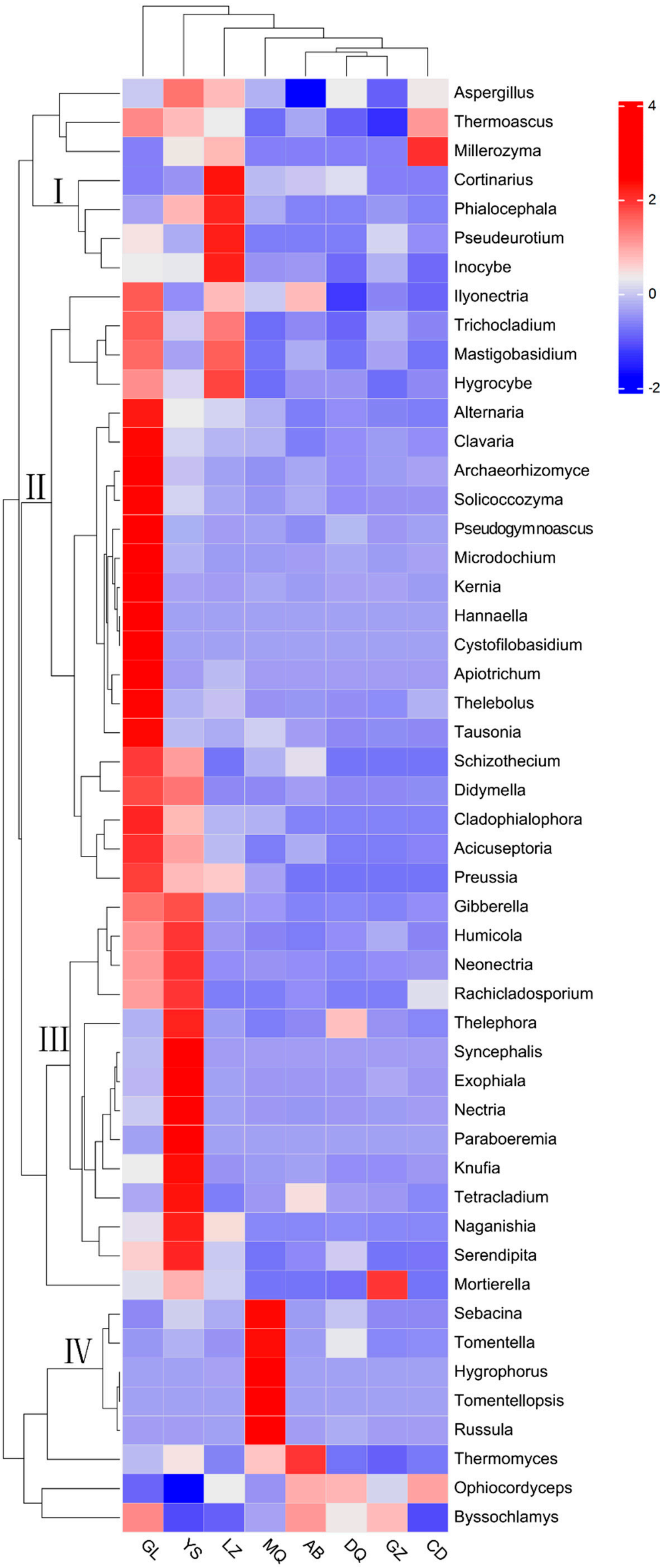

Figure S2. The heatmap of species composition of the top 50 genera level biclustering of *O. sinensis* abundance in different habitats.

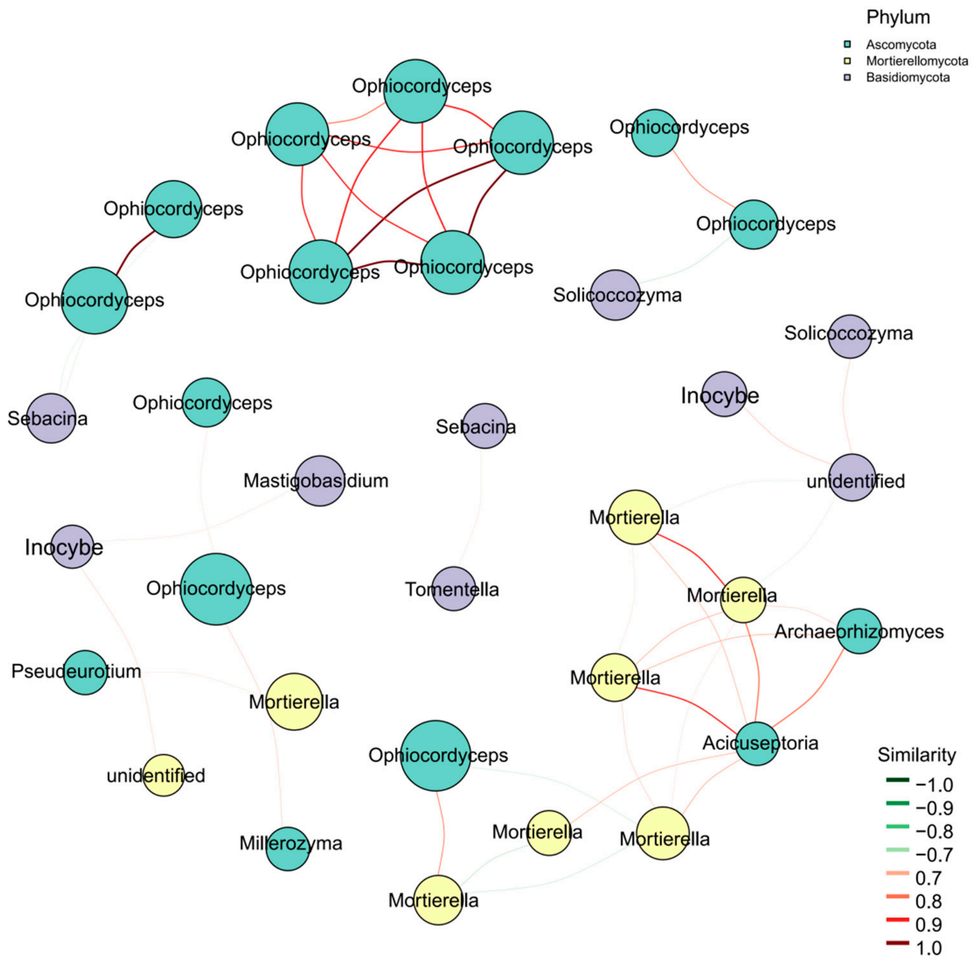

Figure S3. Network diagram of fungal taxa at the phylum level. The red line indicates a positive correlation, and the green line indicates a negative correlation.
